# A systematic review and meta-analysis of health utility values among patients with ischemic stroke

**DOI:** 10.3389/fneur.2023.1219679

**Published:** 2023-09-05

**Authors:** Jiting Zhou, Qiran Wei, Hongfei Hu, Wei Liu, Xin Guan, Aixia Ma, Luying Wang

**Affiliations:** School of International Pharmaceutical Business, China Pharmaceutical University, Nanjing, China

**Keywords:** ischemic stroke, health-related quality of life, utility value, EQ-5D, systematic review

## Abstract

**Purpose:**

Ischemic stroke (IS) has a considerable impact on the health-related quality of life (HRQoL) of patients. A systematic review was conducted to summarize and synthesize the HRQoL reported from IS patients.

**Methods:**

An electronic search was performed in PubMed, Web of Science, ScienceDirect, Embase, and Cochrane Library databases from inception to February 2022 for studies measuring utility values in IS patients. Basic information about the studies, patient characteristics, measurement of the utility values, and utility values were extracted and summarized. Utility values were pooled according to the time of evaluation, and disease severity was classified with modified Rankin Scale (mRS) scores. The quality of the studies was assessed according to key criteria recommended by the National Institute for Health and Care Excellence.

**Results:**

A total of 39 studies comprising 30,853 participants were included in the study. Measured with EQ-5D-3L, the pooled utility values were 0.42 [95% confidential interval (CI): 0.13 to 0.71], 0.55 (95% CI: 0.43 to 0.68), 0.65 (95% CI: 0.52 to 0.78), 0.60 (95% CI: 0.43 to 0.78), and 0.67 (95% CI: 0.60 to 0.74) for patients diagnosed with IS within 1, 3, 6, 12, and 24 months or above among poststroke patients. Four studies reported utility values classified by mRS scores where synthesized estimates stratified by mRS scores ranged from 0.91 (95% CI: 0.85 to 0.97) for patients with an mRS score of 1 to−0.04 (95% CI:−0.18 to 0.11) for those with an mRS score of 5. As for the health dimension profiles, usual activity was the most impacted dimension, while self-care was the least impacted one.

**Conclusion:**

This study indicated that the utility values in IS patients kept increasing from stroke onset and became relatively stabilized at 6 months poststroke. Health utility values decreased significantly as mRS scores increased. These results facilitate economic evaluations in utility retrieval and selection. Further exploration was required regarding the factors that affect the HRQoL of IS patients.

## 1. Introduction

Stroke continues to be one of the leading causes of death and disability worldwide; with 12.2 million strokes that occurred in 2019, ischemic stroke (IS) accounted for 62.4% (1). The disease burden of IS increases continuously in China, where the number of incident cases was estimated to reach 2.8 million in 2019 (2). Despite the advances in early management and secondary prevention, deaths from stroke have increased by 43.0% over the last three decades (1, 3, 4). Additionally, patients who survive IS often experience long-term disability (5), cognitive impairment (6), and emotional problems (7), leading to compromised health-related quality of life (HRQoL).

HRQoL can be defined as how well a person functions in their life and his or her perceived wellbeing in physical, mental, and social domains of health and can be presented using utility (8), which ranges from 0 to 1, where 0 represents death and 1 represents perfect health, and a negative utility value represents health states that are worse than death (9). There are various methods to directly and indirectly measure utility values; these methods can be performed among patients, their caregivers, or the general public. Direct evaluation methods such as time trade-off (TTO), standard gamble (SG), and rating scale (RS) elicit values directly from respondents who make their assignment with respect to specific health states or are required to report their preferences toward some hypothetical scenarios. However, these evaluation methods take more time and may involve problems regarding cognitive understanding and interviewer effects (10, 11). Indirect valuation methods such as the EuroQol 5-dimensional (12) (EQ-5D), Short Form 6 Dimensions (13) (SF-6D), 15D (14), and Health Utility Value Index (15) (HUI) are questionnaires that are easier to administer and thus can be included as a part of clinical trials or routine follow-up without increasing respondent burden (16). In the questionnaires, respondents specify their health states in multiple dimensions, and the questionnaire responses are then converted to utility values by means of “tariffs” (17). The “tariffs” are obtained from previous studies in which values for possible health states were elicited from the general population using methods such as TTO (12, 17).

Utility can also be applied to estimate quality-adjusted life years (QALYs) gained for cost-effectiveness analysis by multiplying by the time of survival in a certain health state. With the launch of new medical techniques, the use of cost-effective analysis to compare the potential benefits, harms, and costs between new interventions and existing interventions is an important technique for healthcare decision-makers and has been widely adopted in many countries to help better allocate medical resources (18).

Given the important role of health utility values in summarizing HRQoL and supporting cost-effectiveness analysis, this systematic review aimed to identify and summarize studies reporting utility values in IS and provide the pooled utility values of the IS population at different times of measurement and disease severities.

## 2. Methods

This systematic review was performed in accordance with the Preferred Reporting Items for Systematic Reviews and Meta-analyses (PRISMA) statement (19).

### 2.1. Search strategy

The PubMed, Embase, Cochrane Library, Web of Science, and ScienceDirect databases were searched from inception to February 2022. Search terms included “ischemic stroke,” “ischaemic stroke,” “patient reported outcomes,” “quality of life,” “QoL” and “HRQoL.” Detailed information on search items in the abovementioned database is shown in Supplementary Table 1.

### 2.2. Inclusion and exclusion criteria

According to the PICOS framework, both clinical trials and observational studies that reported outcomes on utility values in IS patients were included. There were no restrictions on interventions and comparators. In order to decrease the heterogeneity and uncertainty, studies were excluded if they met any of the following criteria: (a) only abstracts or studies with full-text unavailable; (b) systematic review; (c) economic evaluation; (d) not published in English; (e) reported utility values for a mixed cohort of patients with IS and hemorrhagic stroke; and (f) non-original study that did not provide additional information on health utility.

### 2.3. Data extraction

The characteristics of the included studies were extracted independently by two reviewers, with disagreements resolved through discussion or a third reviewer. The following data were extracted from the included studies: study characteristics (year of publication, country or region, study design, sample size, and intervention/grouping), demographic characteristics of patients (age and gender), methodology of HRQoL measurement (survey method, evaluation time, and tariff), and utility values.

### 2.4. Data analysis and synthesis

To observe the long-term changes in utility values in the IS population, mean utility values elicited with the EQ-5D were synthesized by meta-analysis according to the appropriate time of evaluation. Since few studies reported the mean utility values using EQ-5D-5L, only mean utility values measured using EQ-5D-3L were pooled. Considering the short duration of stay, days from discharge were regarded as days from poststroke. Additionally, the difference in synthesized utility values between each evaluation time was compared with minimally important differences (MIDs) in the EQ-5D-3L in stroke. The MID in the EQ-5D was 0.08 to 0.12 (20), and a 0.1-point increase or decrease in utility was considered an important change in our study. Furthermore, the utility values stratified by the modified Rankin Scale (mRS) were also pooled to describe utility weights for individuals with different mRS scores. The mRS is a commonly used clinician-reported scale that assesses changes in disability after stroke, with scores ranging from 0 to 6 (21).

Notably, this study aimed to synthesize utility values for IS population, and utility values derived from patients who entered the randomized controlled studies and received the specific treatments (e.g., intensive lipid rather than guideline lipid) or participated in some therapeutic programs (e.g., home rehabilitation program, which was a home-based exercise program provided by a physical therapist) tend to generate better health states with higher utility values and could not represent the general population. Similarly, the utility values for IS patients with specific poststroke complications (e.g., spasticity) were not included. Pooled utility values of compared groups from clinical trials were also excluded. For multiple publications from the same study population, the article that reported utility values appropriate for meta-analysis or covered a larger sample size was included. For any study reporting utility values applying diverse tariffs from multiple countries, to eliminate the additive effect, only the utility value calculated using investigators' country-specific tariff was included in the meta-analysis.

If the standard deviation (SD) around the mean utility value was not available in the article, estimations from the standard error or 95% confidential interval (CI) were applied. The heterogeneity among the included studies was assessed using the *I*^2^ statistic, and fixed effects models were employed if the value of *I*^2^ was smaller than 50%; otherwise, random effects models were used. The meta-analysis was conducted in R software version 4.2.1 using the “meta” package.

### 2.5. Quality assessment

Since there were no agreed criteria specific for assessing the quality of utility studies, we assessed the quality of the included studies based on the criteria recommended in the National Institute for Health and Clinical Excellence guidance on a systematic review of utility values (22) and additional two criteria (uncertainty measurement and appropriateness of tariff) applied in the study by Mok et al. (23).

## 3. Results

### 3.1. Study selection

**Figure 1:**
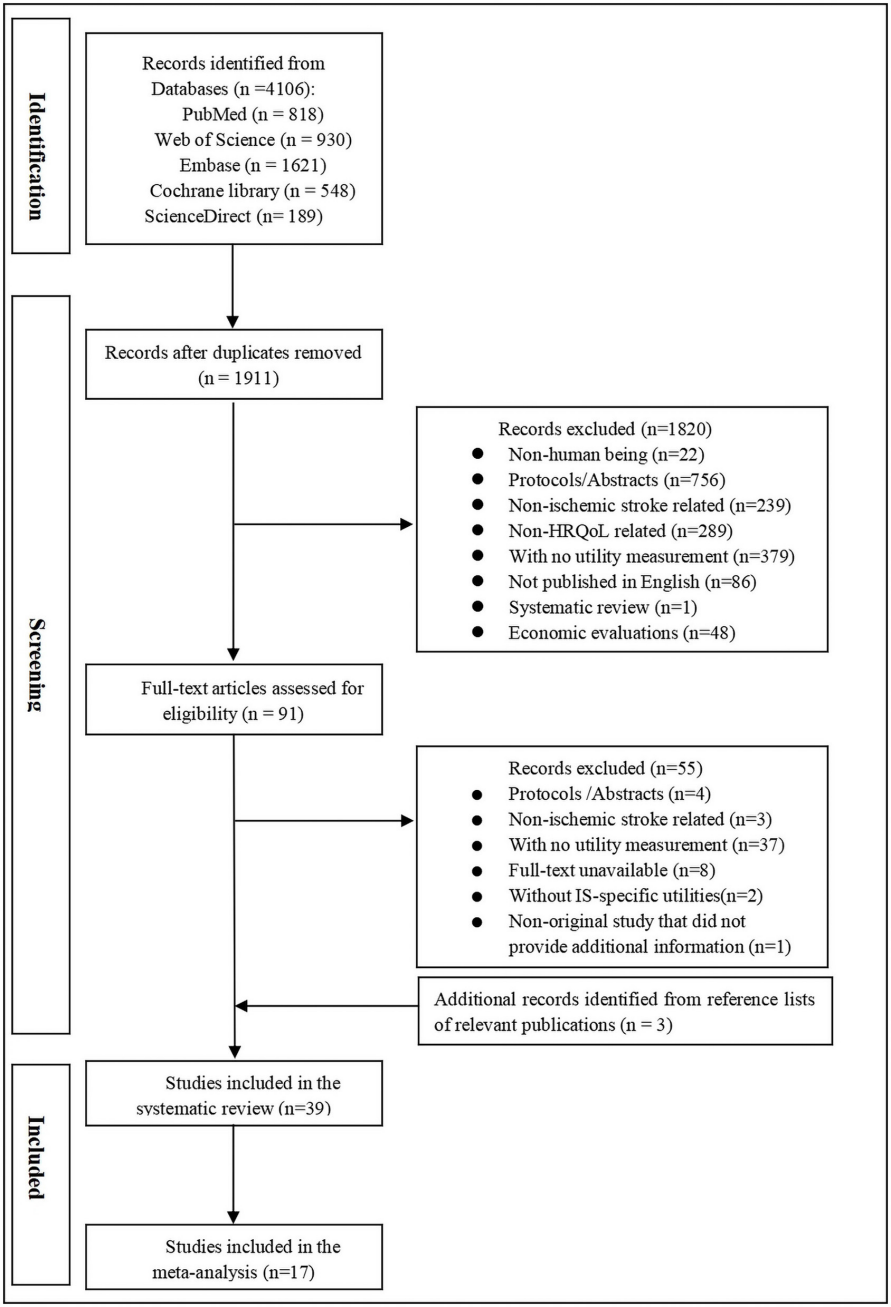
Flowchart of the study inclusion process.

The flowchart of study selection and the inclusion process is presented in Figure 1. Our search initially identified 4,106 references. After removing duplicates, 1,911 records were further screened by titles and abstracts. Finally, 91 articles were subject to full-text screening, where 55 were excluded mostly because they did not involve utility evaluation. Three additional studies were identified from the reference lists of relevant publications. A total of 39 studies were included, and 17 studies were selected for meta-analysis.

### 3.2. Basic characteristics of the included studies

The basic characteristics of the included studies are summarized in Table 1. A total of 39 identified studies were published between 1999 (24) and 2022 (25), with most of studies (*n* = 8) (26–33) published in 2017. The included studies covered different regions around the world. Five of the studies were multinational (26, 31, 34–36), 18 studies in Europe (24, 27, 28, 30, 32, 33, 37–48), 8 studies in Asia (25, 49–55), 7 studies in North America (29, 56–61), and 1 study in Australia (62). In terms of study design, 22 of the 39 (56.4%) studies were observational, while the rest were randomized controlled studies to assess the effectiveness of treatment. In the randomized controlled studies, endovascular treatment (EVT) was the most common therapy (25, 28, 31–33, 35, 43, 54). Other treatments included a rehabilitation program, alteplase, citicoline, intravenous tissue plasminogen activator (t-PA), lipid management, and blood pressure management. Studies included 30,853 participants (adjustment has been made for the overlapping populations), and the sample size ranged from 11 to 4,016. The average or median age of the study population was >60 years in most of the studies. The proportion of males ranged from 36.0 to 81.6%.

**Table 1:** Basic characteristics of the included studies.

**Study**	**Country/ region**	**Study design**	**Intervention/ grouping**	**Sample size**	**Male (%)**	**Age (mean ±sd)**
Hallan et al. (24)	Norway	Obs	a: minor stroke b: major stroke	158	47.0	NR
Pickard et al. (56)	Canada	Obs	NR	124	53.0	68.3 ± 14.6
Haacke et al. (37)	Germany	Obs	NR	54	48.0	NR
Chaiyawat et al. (49)	Thailand	RCT	a: home rehabilitation program b: usual care	a: 30 b: 30	a: 47.0 b: 43.0	a: 67.0 ± 10.0 b: 66.0 ± 11.0
Lee et al. (50)	Taiwan	Obs	a: lacunar infarction b: non-lacunar infarction	a: 170 b: 263	a: 62.9 b: 63.5	a: 67.7 ± 10.4 b: 63.7 ± 13.1
Chiayawat et al. (51)	Thailand	RCT	a: home rehabilitation program b: usual care	a: 30 b: 30	a: 47.0 b: 43.0	a: 67.0 ± 10.0 b: 66.0 ± 11.0
Naess et al. (38)	Norway	Obs	NR	328	63.0	67.7
Luengo-fernandez et al. (39)	UK	Obs	NR	404	NR	NR
The IST-3 group (34)	Multinational	RCT	a: alteplase + standard care b: standard care	a: 1,169 b: 1,179	a: 49.0 b: 49.0	NR
Bushnell et al. (58)	USA	Obs	NR	1,370	53.7	65.0^*^(56.0–75.0)^#^
Kelly et al. (57)	USA	Obs	Hemicraniectomy	11	36.0	55.0^*^(42.0-62.0)^#^
Gillard et al. (59)	USA	Obs	a: patients with spasticity b: patients without spasticity	a: 54 b: 274	a: 54.0 b: 51.0	a: 59.7 ± 14.1 b: 67.1 ± 13.5
Alvarez-Sabín et al. (40)	Spain	RCT	citicoline/usual treatment	163	50.9	67.5 ± 10.7
Chang et al. (52)	Korea	Obs	NR	2,289	62.2	65.7 ± 12.4
Rangaraju et al. (35)	Multinational	RCT	EVT + intravenous t-PA/intravenous t-PA alone	423	NR	NR
Sand et al. (41)	Norway	Obs	a: with vision problem b: with normal vision	a: 83 b: 244	a: 55.4 b: 65.2	a: 71.8 ± 14.3 b: 66.5 ± 12.4
Ali et al. (26)	Multinational	RCT	NR	3,858	NR	67.5 ± 12.5
Bath et al. (27)	UK	RCT	a: intensive lipids b: guideline lipids	a: 39 b: 38	a: 76.9 b: 81.6	a: 74.2 ± 6.4 b: 74.4 ± 6.9
Dávalos et al. (28)	Spain	RCT	a: EVT + medical treatment b: medical treatment	a: 103 b: 103	a: 53 b: 52	a: 65.7 ± 11.3 b: 67.2 ± 9.5
Katzan et al. (29)	USA	Obs	NR	3,283	54.0	63.5 ± 14.4
Persson et al. (30)	Sweden	Obs	NR	248	66.0	64.0 ± 11.0
Rangaraju et al. (31)	Multinational	RCT	EVT + intravenous t-PA/intravenous t-PA alone	423	NR	64.0 ± 13.0
Schreuders et al. (32)	Netherlands	RCT	a: EVT + usual care b: usual care	457	41.1	66.0^*^(56.0-76.0)^#^
van den berg et al. (33)	Netherlands	RCT	a: EVT + usual care b: usual care	a: 194 b: 197	a: 57.2 b: 59.9	a: 65.9^*^(55.8–76.2)^#^ b: 65.5^*^(56.6–76.6)^#^
Chung et al. (53)	Korea	Obs	NR	991	65.6	64.3 ± 12.0
Dijkland et al. (43)	Netherlands	RCT	a: EVT + usual care b: usual care	a: 233 b: 267	a: 58.0 b: 59.0	a: 65.8^*^(54.5–76.0)^#^ b: 65.7^*^(55.5–76.4)^#^
Winter et al. (42)	Germany	Obs	a: with poststroke epilepsy b: without poststroke epilepsy	a: 23 b: 351	a: 56.2 b: 57.0	a: 67.0 ± 8.4 b: 69.0 ± 4.9
Dewilde et al. (44)	Belgium	Obs	NR	539	58.9	68.7 ± 12.9
Oemrawsingh et al. (45)	Netherlands	Obs	NR	1,022	57.0	74.0^*^(64.0–82.0)^#^
Chen et al. (36)	Multinational	RCT	Standard-dose/low-dose rt-PA/intensive BP lowering/guideline-recommended BP lowering	4,016	62.4	66.1
Jarosławski et al. (46)	Poland	Obs	NR	171	47.7	70.5
Willeit et al. (47)	Austria	RCT	a: STROKE-CARD care b: standard care	a: 1,438 b: 711	a: 59.0 b: 59.0	a: 69.0 ± 14.0 b: 70.0 ± 13.0
Yang et al. (54)	China	RCT	a: EVT b: alteplase + EVT	a: 327 b: 329	a: 57.8 b: 55.0	a: 69.0^*^ (61.0–76.0)^#^ b: 69.0^*^ (61.0–76.0) ^#^
Parameshwaran et al. (62)	Australia	Obs	EVT	145	57.0	70.0 ± 13.3
Romano et al. (60)	USA	Obs	NR	1,765	58.0	65.0 ± 14.0
Schneider et al. (48)	Estonia	Obs	NR	352	63.1	48.8^*^
She et al. (55)	China	RCT	NR	1,714	63.4	61.4 ± 9.7
Sucharew et al. (61)	USA	Obs	NR	294	48.0	70.0^*^ (60.0–79.0)^#^
Zhang et al. (25)	China	RCT	a: with anxiety/depression b: without anxiety/depression	a: 289 b: 226	a: 61.3 b: 51.8	a: 66.8 ± 11.5 b: 67.5 ± 12.7

^*^Median; ^#^Interquartile range. Obs, observational; RCT, randomized controlled trial; sd, standard deviation; EVT, endovascular treatment; t-PA, tissue plasminogen activator; BP, blood pressure; NR, not reported.

### 3.3. Utility score evaluation methods

The evaluation methods are presented in Table 2. For methods applied, three studies (40, 42, 46) did not specify the survey method, while nine studies (25, 28, 34, 36, 44, 45, 53, 54, 61) used more than one method. The survey methods included telephone interviews (*n* = 16, 41.0%), face-to-face interviews (*n* = 11, 28.2%), questionnaires during the follow-up visit (*n* = 10, 25.6%), and postal questionnaires (*n* = 4, 10.3%). As for the respondents, only one study involved a normal population (24), in which they were asked to imagine their preference for certain scenarios as IS survivors. Among the studies that reported specific information on respondents, the percentage of proxies in the reported studies ranged from 12 to 56%. Regarding the utility score elicitation method, only one study used direct methods where TTO, SG, and RS were adopted simultaneously (24). For the indirect methods, the vast majority of studies (*n* = 37) used the EQ-5D, most of which (*n* = 31) used the EQ-5D-3L, 4 studies used EQ-5D-5L, and 2 studies did not mention the EQ-5D-3L/EQ-5D-5L version. In addition, the time point of evaluation for IS patients in the included studies ranged from the stroke onset to 7 years poststroke. A total of 3, 6, 12, and 24 months after stroke/discharge were the most frequently adopted evaluation time points. For tariffs to calculate utility values, 20 studies did not specify the tariffs, and 2 of 5 multinational studies (26, 34) used tariffs from multiple countries of the study population.

**Table 2:** Utility evaluation methods and results.

**Study**	**Survey method**	**Valuation instrument**	**Tariff**	**Valuation time**	**Respondents**	**Utility values**	**EQ-VAS**
Hallan et al. (24)	Interview (supported by an interactive computer program)	SG, TTO, Direct scaling	NA	NR	Healthy people: 42% Non-stroke patients: 32% Stroke survivors: 26%	SG: a: 0.91^*^, b: 0.61^*^ TTO: a: 0.88^*^, b: 0.51^*^ Direct scaling: a: 0.71^*^, b: 0.31^*^	NA
Pickard et al. (56)	Self-administered questionnaire	EQ-5D-3L, HUI3	UK	Baseline (after the acute phase but before discharge) 1 month, 3 months, 6 months	Patients and proxies answered questionnaires separately	Patients: EQ-5D-3L: 0.31 ± 0.38; HUI3: 0.21 ± 0.30 EQ-5D-3L: 0.55 ± 0.36; HUI3: 0.42 ± 0.36 EQ-5D-3L: 0.61 ± 0.30; HUI3: 0.45 ± 0.34 EQ-5D-3L: 0.62 ± 0.34; HUI3: 0.44 ± 0.37	61 ± 17 64 ± 19 69 ± 17 70 ± 18
Haacke et al. (37)	Face-to-face interview	EQ-5D-3L, HUI2, HUI3	NR	4 years after stroke	Patients	• EQ-5D-3L:0.68 ± 0.33 • HUI2: 0.61 ± 0.24 • HUI3: 0.36 ± 0.38	56.74 ± 22.10
Chaiyawat et al. (49)	Face-to-face interview	EQ-5D-3L	NR	Baseline 3 months after stroke	Patients	• a:−0.14 ± 0.08; b:−0.11 ± 0.13 • a: 0.88 (SE 0.02); b:0.53 (SE 0.02)	NR
Lee et al. (50)	Interview	EQ-5D-3L	USA, UK	4.0 to 5.1 years after stroke	Patients	a: 0.8 ± 0.2 b: 0.7 ± 0.3	NR
Chiayawat et al. (51)	Face-to-face interview	EQ-5D-3L	NR	2 years after stroke	Patients	a: 0.9 ± 0.02 b: 0.7 ± 0.04	NR
Naess et al. (38)	Postal survey	EQ-5D-3L 15D	NA	at least 6 months after stroke	Patients: 80% Proxy: 20%	EQ-5D-3L: 0.70 ± 0.30 15D: 0.82 ± 0.14	66 ± 21
Luengo-fernandez et al. (39)	Interview	EQ-5D-3L	UK	1 month, 6 months 1 year, 2 years, 5 years	Patients	0.64 ± 0.33 0.70 ± 0.29 0.70 ± 0.27 0.66 ± 0.29 0.67 ± 0.31	NR
The IST-3 group (34)	Telephone interview, Postal survey	EQ-5D-3L	UK and other European tariff	18 months after stroke	Patients: 44% Proxy: 56%	• a: 0.55 (SE 0.015) • b: 0.50 (SE 0.016)	NR
Bushnell et al. (58)	Telephone interview	EQ-5D-3L	USA	3 months post-discharge 1 year post-discharge	Patients	• 0.83^*^ (0.76–1.00)^#^ • 0.83^*^ (0.74–1.00)^#^	NR
Kelly et al. (57)	Self-administered questionnaire	EQ-5D-3L	NR	3 months after hemicraniectomy 9 months post-hemicraniectomy	• Patients without assistance: 45% • Patients with assistance: 55%	0.33^*^ (0.12–0.51)^#^ 0.69^*^ (0.40–0.71)^#^	NR
Gillard et al. (59)	Telephone interview	EQ-5D-3L	USA	3 months after stroke 1 year after stroke 2 years after stroke	Patients	a: 0.59 (SE 0.03) b:0.71 (SE 0.11) a: 0.60 (SE 0.03) b:0.73 (SE 0.01) a: 0.64 (SE 0.04) b:0.72 (SE 0.02)	NR
Alvarez-Sabín et al. (40)	NR	EQ-5D-3L	NR	2 years after stroke	Patients	0.63 ± 0.28	64.4 ± 25
Chang et al. (52)	Face-to-face interview	EQ-5D-3L	NR	6 months after stroke	Patients	0.82 ± 0.19	NR
Rangaraju et al. (35)	Self-administered questionnaire	EQ-5D-3L	USA	3 months after randomization	Patients	• NIHSS 0-4: 0.86 ± 0.16 NIHSS 5-11: 0.77 ± 0.18 • NIHSS 12-19: 0.59 ± 0.26 • NIHSS ≥20: 0.52 ± 0.26	NR
Sand et al. (41)	Postal survey	EQ-5D-3L,15D	NR	At least 6 months poststroke	Patients	EQ-5D-3L: a: 0.62^*^(0.23–0.73)^#^, b:0.80^*^(0.69–1)^#^ 15D: a: 0.73^*^(0.63–0.82)^#^, b: 0.89 (0.79–0.95) ^#^	NR
Ali et al. (26)	Self-administered questionnaire	EQ-5D-3L	12 countries	3 months after stroke	Patients: 76.4% Proxy: 21.8%	Utility values were reported based on mRS score.	NR
Bath et al. (27)	Telephone interview	EQ-5D-3L	NR	Baseline (3–7 months poststroke event), around 2 years after randomization	Patients	Baseline: 0.8 ± 0.2 (all) 2 years after randomization: a: 0.8 ± 0.2, b:0.7 ± 0.2	Baseline: 72.9 ± 17.6 Follow-up: a:69.0 ± 22.0 b:73.2 ± 14.5
Dávalos et al. (28)	Face-to-face interview, telephone interview	EQ-5D-3L	Spanish	3 months after stroke 6 months after stroke 1 year after stroke	3/6/12 months: Patients: a:82.1%; 63.4%; 83.5% b:82.8%; 65.9%; 77.9%	a: 0.44 ± 0.36, b: 0.34 ± 0.34 a: 0.45 ± 0.36, b: 0.34 ± 0.34 a: 0.46 ± 0.38, b: 0.33 ± 0.33	3 months: a: 60.0 ± 22.0 b:52.2 ± 23.8 6 months: a:59.9 ± 22.8 b:52.3 ± 24.1 1 year: a:63.0 ± 23.9 b:57.0 ± 23.8
Katzan et al. (29)	Self-administered questionnaire	EQ-5D	NR	58^*^(32-258)^#^ days after stroke	Patients	0.79^*^ (0.68-0.84)^#^	NR
Persson et al. (30)	Self-administered questionnaire	SF-6D	UK	7 years after stroke	Patients	0.70 ± 0.12	NA
Rangaraju et al. (31)	Self-administered questionnaire	EQ-5D-3L	USA	3 months after randomization	Patients	0.73 ± 0.24	NR
Schreuders et al. (32)	Telephone interview	EQ-5D-3L	Dutch	3 months after stroke	Patients	a: 0.57^*^ b: 0.39^*^	NR
van den berg et al. (33)	Telephone interview	EQ-5D-3L	NR	2 years after stroke	Patients	a: 0.48 ± 0.40 b: 0.38 ± 0.39	NR
Chung et al. (53)	Self-administered questionnaire, telephone interview	EQ-5D-3L	NR	Baseline (discharge or within 1 month after discharge) 3 months after discharge 6 months after discharge	Patients	0.67 ± 0.21 0.72 ± 0.18 0.73 ± 0.16	69.25 ± 17.52 74.38 ± 13.85 76.54 ± 13.35
Dijkland et al. (43)	Face-to-face interview	EQ-5D-3L	Dutch	3 months after stroke	Patients: 62% Proxy: 38%	Overall: 0.45 ± 0.32 a: 0.50 ± 0.33 b: 0.41 ± 0.31	NR
Winter et al. (42)	NR	EQ-5D-3L	German	Admission 6 months 1 year 2 years	Patients	a: 0.55 ± 0.27, b: 0.59 ± 0.29 a: 0.62 ± 0.36, b: 0.69 ± 0.37 a: 0.51 ± 0.23, b: 0.65 ± 0.19 a: 0.52 ± 0.31, b: 0.66 ± 0.24	a:51.84 ± 10.83 b:54.84 ± 17.93 a:58.34 ± 27.49 b:67.42 ± 20.17 a:56.38 ± 11.24 b:64.77 ± 14.51 a:55.27 ± 10.74 b:64.24 ± 11.44
Dewilde et al. (44)	Self-administered questionnaire, telephone interview	EQ-5D-3L	European	3–36 months after stroke	Patients: 70% Proxy: 30%	Utility values were reported based on mRS score	NR
Oemrawsingh et al. (45)	Face-to-face interview, telephone interview	EQ-5D-5L	Dutch	3 months post-discharge	Patients or proxies	0.65^*^ (0.1-0.83) ^#^	NR
Chen et al. (36)	Face-to-face interview, telephone interview	EQ-5D-3L	UK	3 months after stroke	Patients: 63% Proxy: 37%	0.72 ± 0.37	NR
Jarosławski et al. (46)	NR	EQ-5D-3L	UK, Poland	6-18 months after stroke	Patients	UK standard: 0.51 Polish standard: 0.68	54.4
Willeit et al. (47)	Self-administered questionnaire	EQ-5D-3L	European	12 months after discharge	Patients	a: 0.78^*^ (0.69–1.00) ^#^ b: 0.78^*^ (0.57–1.00) ^#^	NR
Yang et al. (54)	Face-to-face interview, telephone interview	EQ-5D-5L	NR	3 months after stroke	Patients	a: 0.84^*^ (0.48–0.95) ^#^ b: 0.85^*^ (0.26–1.00)^#^	NR
Parameshwaran et al. (62)	Telephone interview	EQ-5D-3L	NR	12 months after EVT	Patients	Utility values were reported based on mRS score	NR
Romano et al. (60)	Telephone interview	EQ-5D-5L	NR	3 months after stroke	• Patients: 76% • Proxies: 12% • Undocumented: 12%	0.85 ± 0.17	77 ± 19
Schneider et al. (48)	Postal survey	EQ-5D-3L	Poland	5.7 years after stroke	Patients	0.71 ± 0.28	NR
She et al. (55)	Face-to-face interview	EQ-5D-3L	China	Within 2 weeks after hospitalization	Patients	0.75 ± 0.23	72.7 ± 15.8
Sucharew et al. (61)	EMR, Telephone interview	EQ-5D	USA	3 months after stroke 6 months after stroke	3/6 months: Patients: 66/72% Proxy: 34%/28%	EMR reviewer: 0.78^*^ (0.60–0.83)^#^, Telephone interviews: 0.81^*^(0.60–0.85) ^#^ EMR reviewer: 0.78^*^ (0.69–0.84), Telephone interviews: 0.83^*^ (0.71–1.00) ^#^	NR
Zhang et al. (25)	Face-to-face interview, telephone interview	EQ-5D-5L	NR	3 months after stroke	Patients	a:0.96^*^ (0.78–1.00)^#^ b:0.57^*^ (0.06–0.85)^#^	NR

The version of EQ-5D in studies that were published before 2011 and was considered EQ-5D-3L (63). ^*^Represents median. ^#^Represents interquartile range. SG, standard gamble; TTO, time trade-off; EQ-5D, EuroQol 5-dimensional; HUI2, Health Utilities Index Mark 2; HUI3, Health Utilities Index Mark 3; NA, not applicable; NR, not reported; SE, standard error; NIHSS, National Institutes of Health Stroke Scale; EMRs, electronic medical records.

### 3.4. Utility results

#### 3.4.1. Utility values classified by the time of evaluation

**Figure 2:**
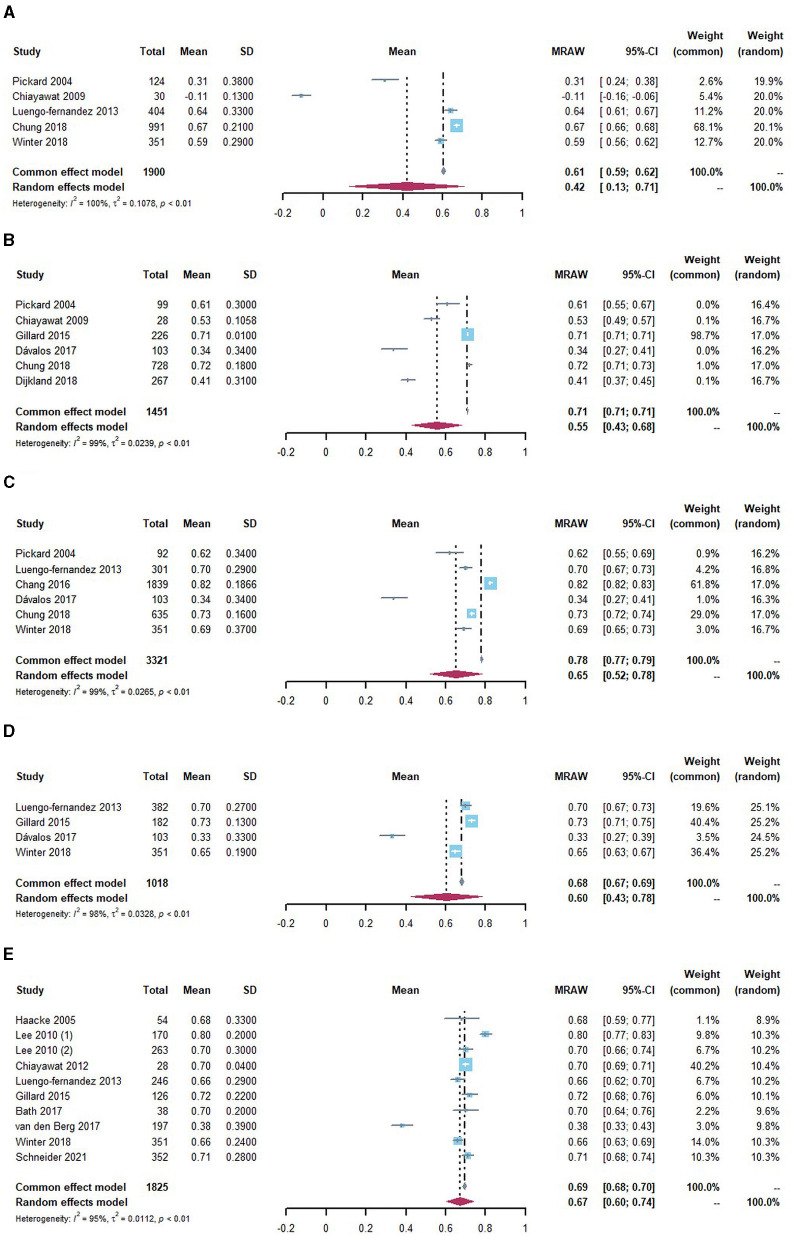
Utility values stratified by evaluation time for IS population **(A)** within 1 month of poststroke, **(B)** at 3 months of poststroke, **(C)** at 6 months of poststroke, **(D)** at 12 months of poststroke, and **(E)** 24 months and above of poststroke.

**Figure 3:**
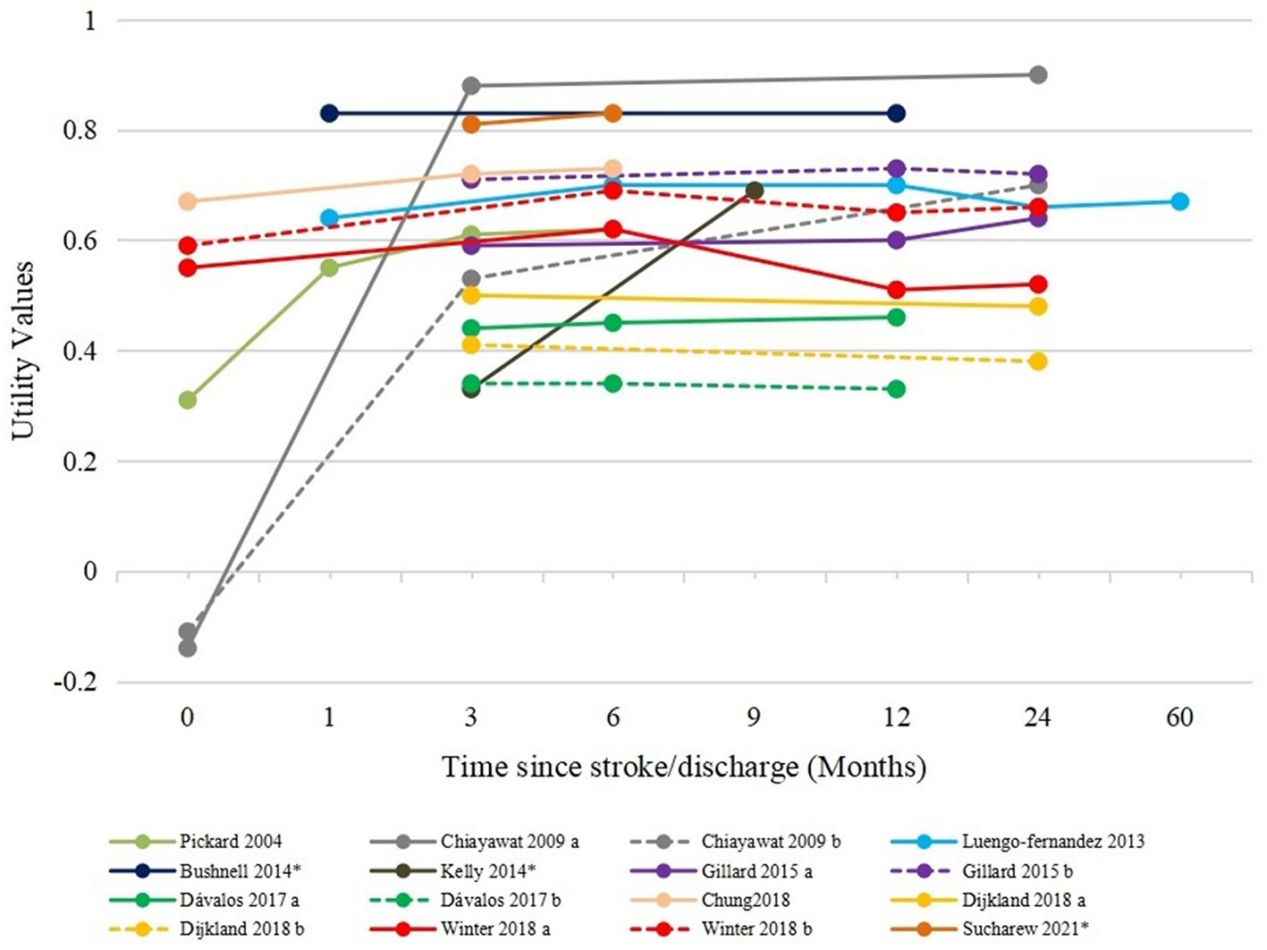
Utility values in longitudinal studies. Chiayawat 2009 a, rehabilitation program; Chiayawat 2009 b, usual care; Gillard 2015 a, patients with spasticity; Gillard 2015 b, patients without spasticity; Dávalos 2017 a, endovascular treatment + medical treatment; Dávalos 2017 b, medical treatment; Dijkland 2018 a, EVT + usual care; Dijkland 2018 b, usual care; Winter 2018 a, patients with poststroke epilepsy; Winter 2018 b, patients with poststroke epilepsy. *Utility values were reported as median values in these studies.

We synthesized the utility values by the baseline (within 1 month after stroke/discharge), 3, 6, 12, and 24 months or above among poststroke patients, as illustrated in Figure 2. For patients at the baseline of stroke onset, the utility values were reported in five studies, ranging from−0.11 to 0.67. Accordingly, the pooled estimate as utility value for the acute stroke phase was 0.42 (95% CI: 0.13 to 0.71), with significant heterogeneity (*I*^2^ = 100%), as presented in Figure 2A. When measured at 3 months after stroke, the utility values were increased and ranged from 0.34 to 0.71 in six studies. The synthesized utility value was 0.55 (95% CI: 0.43 to 0.68). When measured at 6 months after stroke, the utility values ranged from 0.34 to 0.82 in six studies, and the pooled utility value was 0.65 (95% CI: 0.52 to 0.78). A slight decrease could be observed at 12 months poststroke, where the estimated utility values were 0.60 (95% CI: 0.43 to 0.78). The synthesized utility values were 0.67 (95% CI: 0.60 to 0.74) at 24 months and above poststroke, indicating a relatively steady HRQoL among patients. Health utility values kept increasing from stroke onset to 6 months poststroke, and MID could be observed between the baseline and 3 months as well as 3 and 6 months. Therefore, it is concluded that the utility values reached a relatively stable level after 6 months poststroke. The utility values after 6 months were further combined and estimated to be 0.66 (95% CI: 0.59 to 0.72), as presented in Supplementary Figure 1. The trend of change in utility values was similar to that of most of the included longitudinal studies (Figure 3).

#### 3.4.2. Utility values classified by mRS

**Figure 4:**
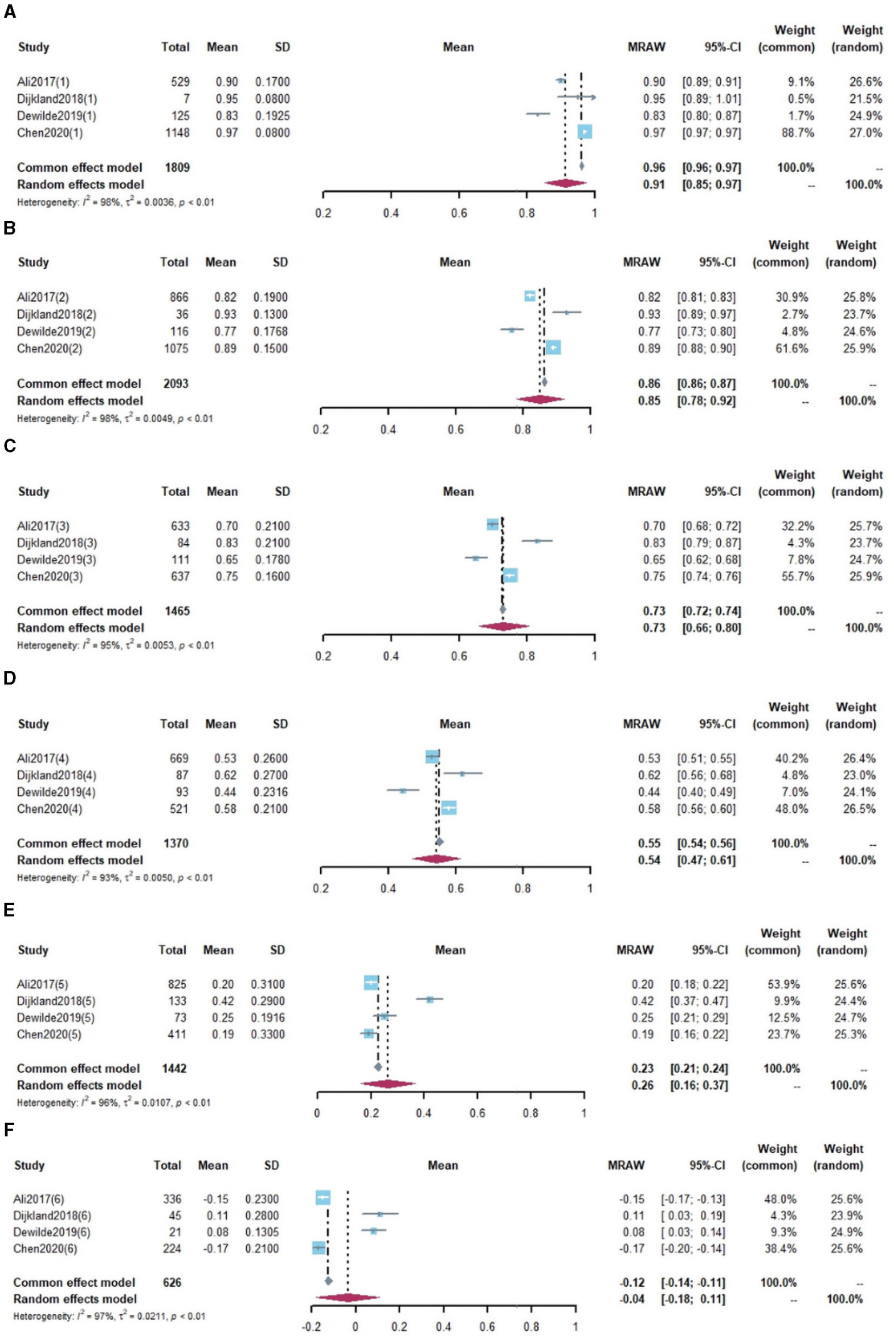
Utility values by modified Rankin Scale for IS population. **(A)** mRS = 0, **(B)** mRS = 1, **(C)** mRS = 2, **(D)** mRS = 3, **(E)** mRS = 4, **(F)** mRS = 5.

Eight studies reported utility values stratified by mRS scores (as shown in Table 3), of which three reported utility values for dichotomized mRS scores (24, 37, 62) (classified as “independence” and “severe disability”) and one study reported the utility index as median values (31). Thus, these four studies were excluded, and the other four studies reported mean values (26, 36, 43, 44) were included in the pooled analysis. With considerable heterogeneity, the pooled effect estimates for mRS scores from 0 to 5 were 0.91, 0.85, 0.73, 0.54, 0.26, and−0.04, respectively, as shown in Figure 4. The MID in utility values was seen between all mRS levels except mRS 0 and mRS 1.

**Table 3:** Utility values classified by the modified Rankin Scale.

**Study**	**mRS = 0**	**mRS = 1**	**mRS = 2**	**mRS = 3**	**mRS = 4**	**mRS = 5**	**mRS = 6**
Hallan et al. (24)	NR	NR	mRS 2-3: 0.91^*^		mRS 4-5: 0.61^*^		NA
Haacke et al. (37)	mRS 0-2 (independence): 0.86			mRS 3-6 (severe disability): 0.44			
Ali et al. (26)	0.90	0.82	0.70	0.53	0.20	−0.15	NA
Rangaraju et al. (31)	1.00^*^	0.84^*^	0.78^*^	0.71^*^	0.44^*^	0.18^*^	NA
Dijkland et al. (43)	0.95	0.93	0.83	0.62	0.42	0.11	0.00
Dewilde et al. (44)	0.83	0.77	0.65	0.44	0.25	0.08	NA
Chen et al. (36)	0.97	0.89	0.75	0.58	0.19	−0.17	NA
Parameshwaran et al. (62)	mRS 0-2: 0.863^*^			mRS 3-5: 0.324^*^			NA

^*^Median. mRS, modified Rankin Scale; NA, not applicable.

#### 3.4.3. Utility values for subgroups

The utility of the IS population is affected by many factors. Some of the studies reported utility values for specific subgroups of IS populations. For example, four studies reported utility values for male and female IS patients, and all suggested that men had better HRQoL as measured by the EQ-5D-3L than women (37, 40, 55, 58). In addition, one study reported utility values stratified by National Institutes of Health Stroke Scale (NIHSS) scores, which was another scale for disease severity. It was indicated that utility values at 3 months for NIHSS 0–4, NIHSS 5–11, NIHSS 12–19, and NIHSS ≥ 20 were 0.86, 0.77, 0.59, and 0.52, respectively (35).

Poststroke complications also played an important role in affecting utility values, and it was reported that IS patients with poststroke spasticity had lower scores on the EQ-5D (0.63 vs. 0.71) (59). Similarly, patients with poststroke epilepsy reported worse HRQoL in the long term (42).

Moreover, the utility values were affected by the valuation instrument. In four studies that reported instrument-specific utility values, the utility values elicited by EQ-5D were lower than those elicited by 15D (38, 41) but higher than those elicited by HUI3 (37, 56). Additionally, the utility was mediated by respondents due to differences in the perception of HRQoL between patients and their proxies, in two studies that reported the utility of specific respondents, both suggested that the utility was slightly lower for proxies than for patient self-assessments (56, 61).

### 3.5. Responses to EQ-5D dimensions

**Figure 5:**
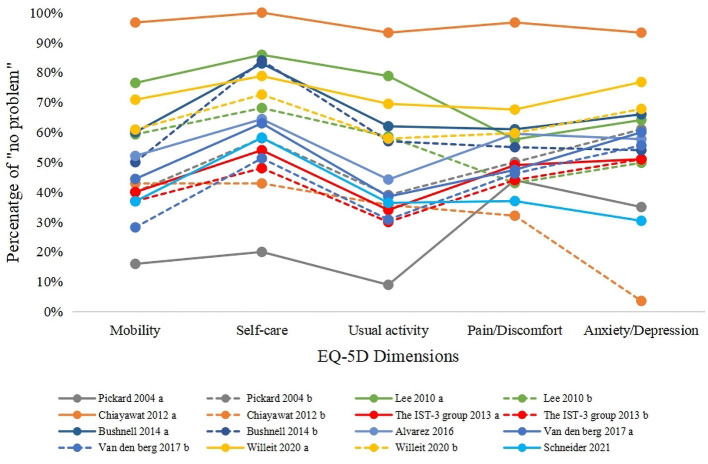
Responses to EQ-5D dimensions. Pickard 2004 a, within 2 weeks of stroke (baseline); Pickard 2004 b, 6 months after baseline; Lee 2010 a, lacunar infarction; Lee 2010 b, non-lacunar infarction; Chiayawat 2012 a, rehabilitation program; Chiayawat 2012 b, usual care; The IST-3 group a, alteplase + standard care; The IST-3 group b, standard care; Bushnell 2014 a, male; Bushnell 2014 b, female; Van den berg 2017 a, endovascular treatment plus usual care; Van den berg 2017 b, usual care.

Nine studies (16 groups) reported the responses to EQ-5D dimensions. The overall information on responses to five dimensions is illustrated in Figure 5. Given the differences in baseline characteristics, disease severity of the study population, and evaluation time, the proportion of patients reporting “no problems” in each dimension varied greatly. Nevertheless, by comparing the dimensions, 12 groups suggested that the proportion of patients who reported “no problems” in the self-care dimension was higher than that of other dimensions. Additionally, six groups showed that the proportion of patients who reported “no problems” in the usual activity dimension was lower than that of other dimensions. In general, the most impaired health dimension was usual activity, while self-care was the least impacted dimension. Anxiety/depression, pain/discomfort, and mobility dimensions were moderately affected.

### 3.6. Quality assessment

The quality assessment of the included studies is presented in Supplementary Table 2. Many studies did not provide sufficient information on evaluation methodology or utility results to be assessed by several criteria, such as response rates to instruments and missing data. More specifically, in terms of sample size, the utility values were elicited from fewer than 100 participants in five studies (27, 37, 49, 51, 57). The sample size was smaller within the studies focused on specific patients such as those who underwent hemicraniectomy or who had specific complications. The recruitment and inclusion criteria were given in most of the included studies, and since most of the studies applied restrictions on age, disease severity, medical history, and so on and excluded the most severe patients, an overestimate of utility may be induced.

Regarding the results reporting, 22 studies (56.4%) reported their response rates to utility instruments, and none of them were lower than 60%. In addition, in eight included longitudinal studies, only one study (42) did not report information on lost follow-up. However, it remained unclear whether patient characteristics with lost follow-up were similar to those of patients followed up for the rest of the studies indicating a potential risk of bias. Information on missing data was reported in a limited number of studies (*n* = 12), and eight of them stated the techniques of handling missing data, such as multiple imputation (39, 45) and analysis based on only complete data (52, 58). The studies that did not report missing data or the corresponding solution could also cause potential biased estimates.

The measures to elicit utility values were all considered valid in all included studies because they all used well-established instruments. For the uncertainty measurements, most of the studies reported SD or interquartile range (IQR) as uncertain estimates, and only two studies (31, 62) reported median utility values without uncertain estimates. Information on tariffs remained unclear in more than 35% of the included studies, and three studies (48, 50, 56) might apply inappropriate tariffs that did not match the country/region of origin of the HRQoL respondents mainly because localized tariffs were not established at the time their studies were conducted.

## 4. Discussion

HRQoL plays an important role in measuring and assessing the total wellbeing of poststroke patients. This study aimed to summarize the HRQoL among patients with IS by a systematic review of previous studies. It could be found that EQ-5D-3L was still the most frequently used tool for measuring HRQoL in IS. Additionally, the meta-analysis was performed according to different times of evaluation poststroke and mRS scores. Thereafter, the results could not only observe and discuss the long-term changes in preference-based HRQoL of IS but also provide utility within each mRS level, both of which could be applied in future economic evaluations. When pooling according to the time of evaluation, the results showed that the mean utility values across the included studies increased gradually over time and then reached a stable level at 6 months poststroke. This made sense because the health condition of patients was expected to be improved after the treatment even though IS could make them suffer from long-term disability. However, the utility values were consistently lower than the matched non-stroke population in the long-term observation (30, 48). When stratified by mRS levels, the utility estimates significantly declined by increasing the mRS levels, and the utility for mRS 5 was negative in our review, suggesting that patients had health states that were worse than death. The summary of utility values for specific groups and the response to EQ-5D dimensions might also provide additional information to researchers. Specifically, female patients and patients with poststroke complications had worse HRQoL, which suggested that more attention should be paid to these patients in disease management. Moreover, since self-care dimension was relatively less impacted, efforts to improve patients' HRQoL should focus on usual activity, anxiety/depression, pain or discomfort, and mobility dimensions.

While there have been numerous studies of HRQoL in patients with IS, to the best of our knowledge, very few studies have reviewed utility values regarding IS. Previous reviews included hemorrhagic stroke and were published almost 2 decades ago (64–66), which might not be representative of the current perception in HRQoL. The recently published review (67), focusing on observational studies, synthesized utility values by the evaluation time points and found that a great increase in utility values between acute care and < 4-month follow-up, which was consistent with our findings. Moreover, we used an additional time point (2 years and above) for pooled utility values because economic evaluations of cardiovascular disease often include health states for 2-year poststroke (68, 69). In comparison with this review, the present systematic review focuses on a certain type of stroke without much restriction on the study design and therefore provides more applicable evidence. Moreover, our findings serve as an update for a current assessment of the evidence on the HRQoL in IS patients.

The study also has several limitations to be considered. First, there was a significant heterogeneity in reported utility values although we followed a rational approach to pool comparable studies that applied EQ-5D-3L to similar evaluation time or disease severity and employed random effects models. Heterogeneity in utility may be induced by the differences in characteristics of participants, including age, gender, and complications. Treatments that were assigned to patients, evaluation methods, and tariffs would also contribute to heterogeneity. Second, there was a poor representation of studies from Central and South America, so it could affect the generalizability of the combined results for these regions. Third, our study may be subject to selection bias since the included studies were limited to those published in English. However, given that English is widely used and well accepted around the world, such influence may be mitigated. Fourth, with limited applicable mean utility values measured with EQ-5D-5L, only mean values measured with EQ-5D-3L were included in the meta-analysis. Furthermore, with a limited number of studies assessing the utility values for subgroups, subgroup analysis was not feasible.

There are some arrears that require further investigation in future research. For instance, utility values stratified by time since IS were important to economic evaluations of cardiovascular disease, our findings might be useful when there were no suitable or appropriate sources, and future evidence on long-term changes in utility values among IS patients from longitudinal studies could be more reliable. Additionally, utility estimates for IS patients with complications needed further explanation since poststroke complication has a negative influence on utility, but few studies have investigated on this. Furthermore, few studies have focused on the impact of IS on utility for caregivers or families, which has been evidenced as a source of significant burden (70). Finally, future HRQoL studies would be more informative if considering the appropriate sample size, evaluation method, reporting the missing data, uncertainty around utility results, and so on. These could facilitate future research studies, health decision–making, and improvement of health policies by providing high-quality data and evidence.

## 5. Conclusion

IS has a substantial effect on patients' HRQoL. This study provided a comprehensive summary of the characteristics of HRQoL research in IS, synthesized health utility values both at different time poststroke and mRS levels, and assessed the quality of studies. The findings from this study will be informative for HRQoL research, economic evaluations, and health decision-making.

## Data availability statement

The original contributions presented in the study are included in the article/Supplementary material, further inquiries can be directed to the corresponding authors.

## Author contributions

LW and AM contributed to the conception of the study. LW and JZ completed the database search. JZ, QW, HH, and WL conducted the screening, data extraction, and quality assessment, under the guidance of LW, XG, and AM. JZ and LW performed the meta-analysis and interpreted the data. JZ, QW, and LW contributed to the writing and revised the manuscript. All authors approved the final manuscript.
