# Supplementary material for: A systematic review and meta-analysis of health utility values among patients with ischemic stroke
Source: Front Neurol. 2023 Sep 5;14:1219679. doi: 10.3389/fneur.2023.1219679 (PMC10507900; doi:10.3389/fneur.2023.1219679)
Supplement: Supplementary file 1 [file Data_Sheet_1.docx]

Supplementary Material

A Systematic Review and Meta-analysis of Health-Related Quality of Life among Patients with Ischemic Stroke

Jiting Zhou^1^, Qiran Wei^1^, Hongfei Hu^1^, Wei Liu^1^, Xin Guan^1^, Aixia Ma^1*^, Luying Wang^1*^

^1^ School of International Pharmaceutical Business, China Pharmaceutical University, Nanjing, China

*** Correspondence:**

Aixia Ma
Email: aixiama73@126.com

Luying Wang
Email: luying_w@163.com

**Supplementary Table 1.** Search details from databases

| Database | Search item | Results |
| --- | --- | --- |
| PubMed | ("ischaemic stroke*"[Title/Abstract] OR"ischemic stroke*"[Title/Abstract]) AND ("patient reported outcome*"[Title/Abstract] OR "HRQOL"[Title/Abstract] OR "QOL"[Title/Abstract] OR "quality of life"[Title/Abstract]) | 818 |
| Cochrane Library | (("ischaemic Stroke?"):ti,ab,kw OR ("ischemic Stroke?"):ti,ab,kw) AND (("patient reported Outcome?"):ti,ab,kw OR ("HRQOL"):ti,ab,kw OR ("QOL"):ti,ab,kw OR ("quality of life"):ti,ab,kw) | 548 |
| Web of Science | #1TI=("ischaemic stroke*" OR "ischemic stroke*")  #2AB=("ischaemic stroke*" OR "ischemic stroke*")  #3TI=("patient reported outcome*" OR "HRQoL" OR "QoL"OR"quality of life")  #4AB=("patient reported outcome*" OR "HRQoL" OR "QoL"OR"quality of life") (#1OR#2) AND #5(#1OR#2)  #6(#3OR#4)  #7(#5AND#6) | 930 |
| ScienceDirect | ("ischemic stroke?" OR "ischaemic stroke?") AND ("quality of life" OR "patient reported outcome?" OR "HRQOL" OR "QOL") | 189 |
| Embase | #1 'patient reported outcome*':ti,ab,kw OR hrqol:ti,ab,kw OR qol:ti,ab,kw OR 'quality of life':ti,ab,kw  #2 'ischemic stroke*':ti,ab,kw OR 'ischaemic stroke*':ti,ab,kw  #3 #1 AND #2 | 1621 |

**Supplementary Table 2.** Results of quality assessment

| **NICE Criteria** | | | | | | | |  | |
| --- | --- | --- | --- | --- | --- | --- | --- | --- | --- |
| **Study** | **Sample size** | **Selection and recruitment** | **Inclusion and exclusion criteria** | **Response rates to instrument** | **Loss to follow-up** | **Missing data** | **Appropriateness of measure** | **Uncertain measurement** | **Appropriateness of tariff use** |
| Hallan1999 [1] | Yes | Yes | Yes | Yes | NA | Unclear | Yes | Yes | NA |
| Pickard 2004 [2] | Yes | Yes | Yes | Yes | Yes | No | Yes | Yes | No |
| Haacke 2005 [3] | No | Yes | Yes | Unclear | NA | Unclear | Yes | Yes | Yes |
| Chaiyawat 2009 [4] | No | Yes | Yes | Yes | Yes | Unclear | Yes | Yes | NA |
| Lee 2010 [5] | Yes | Yes | Yes | Yes | NA | Unclear | Yes | Yes | No |
| Chiayawat 2012 [6] | No | Yes | Yes | Yes | NA | Unclear | Yes | Yes | NA |
| Naess 2012 [7] | Yes | Yes | Yes | Yes | NA | Unclear | Yes | Yes | NA |
| Luengo-Fernandez 2013 [8] | Yes | Yes | Unclear | Yes | Yes | Yes | Yes | Yes | Yes |
| The IST-3 group 2013 [9] | Yes | Yes | Yes | Yes | NA | Yes | Yes | Yes | Yes |
| Bushnell 2014 [10] | Yes | Yes | Yes | Unclear | NA | Yes | Yes | Yes | Yes |
| Kelly 2014 [11] | No | Yes | Yes | Unclear | Yes | Unclear | Yes | Yes | NA |
| Gillard 2015 [12] | Yes | Yes | Yes | Unclear | No | Unclear | Yes | Yes | Yes |
| Alvarez 2016 [13] | Yes | Yes | Yes | Unclear | NA | Unclear | Yes | Yes | Yes |
| Chang 2016 [14] | Yes | Yes | Yes | Unclear | NA | Yes | Yes | Yes | NA |
| Rangaraju 2016 [15] | Yes | Yes | Yes | Unclear | NA | Unclear | Yes | Yes | Yes |
| Sand 2016 [16] | Yes | Yes | Yes | Yes | NA | No | Yes | Yes | Yes |
| Ali 2017 [17] | Yes | Yes | Unclear | Unclear | NA | Yes | Yes | Yes | Yes |
| Bath2017 [18] | No | Yes | Yes | Unclear | Yes | Unclear | Yes | Yes | NA |
| Dávalos2017 [19] | Yes | Yes | Yes | Yes | Yes | Yes | Yes | Yes | Yes |
| Katzan 2017 [20] | Yes | Yes | Unclear | Unclear | NA | Unclear | Yes | Yes | NA |
| Persson 2017 [21] | Yes | Yes | Yes | Unclear | NA | No | Yes | Yes | No |
| Rangaraju 2017 [22] | Yes | Yes | Yes | Unclear | NA | Unclear | Yes | Yes | Yes |
| Schreuders 2017 [23] | Yes | Yes | Yes | Yes | NA | Unclear | Yes | No | Yes |
| Van den berg 2017 [24] | Yes | Yes | Yes | Yes | NA | Yes | Yes | Yes | Yes |
| Chung 2018 [25] | Yes | Yes | Yes | Yes | NA | Unclear | Yes | Yes | NA |
| Dijkland 2018 [26] | Yes | Yes | Yes | Yes | NA | Unclear | Yes | Yes | Yes |
| Winter 2018 [27] | Yes | Yes | Yes | Unclear | Unclear | Unclear | Yes | Yes | NA |
| Dewilde 2019 [28] | Yes | Yes | Yes | Unclear | NA | Unclear | Yes | Yes | Yes |
| Oemrawsingh 2019 [29] | Yes | Yes | Yes | Unclear | NA | Yes | Yes | Yes | Yes |
| Chen 2020 [30] | Yes | Yes | Yes | Yes | NA | Unclear | Yes | Yes | Yes |
| Jarosławski 2020 [31] | Yes | Yes | Yes | Unclear | NA | Unclear | Yes | Yes | Yes |
| Willeit 2020 [32] | Yes | Yes | Yes | Yes | NA | Unclear | Yes | Yes | Yes |
| Yang 2020 [33] | Yes | Yes | Yes | Yes | NA | Unclear | Yes | Yes | NA |
| Parameshwaran 2021 [34] | Yes | Yes | Yes | Yes | NA | Unclear | Yes | No | NA |
| Romano 2021[35] | Yes | Yes | Yes | Unclear | NA | Unclear | Yes | Yes | NA |
| Schneider 2021 [36] | Yes | Yes | Yes | Yes | NA | Unclear | Yes | Yes | Yes |
| She 2021 [37] | Yes | Yes | Yes | Yes | NA | Unclear | Yes | Yes | Yes |
| Sucharew 2021 [38] | Yes | Yes | Yes | Yes | Yes | No | Yes | Yes | Yes |
| Zhang2022 [39] | Yes | Yes | Yes | Yes | NA | Unclear | Yes | Yes | NA |

**
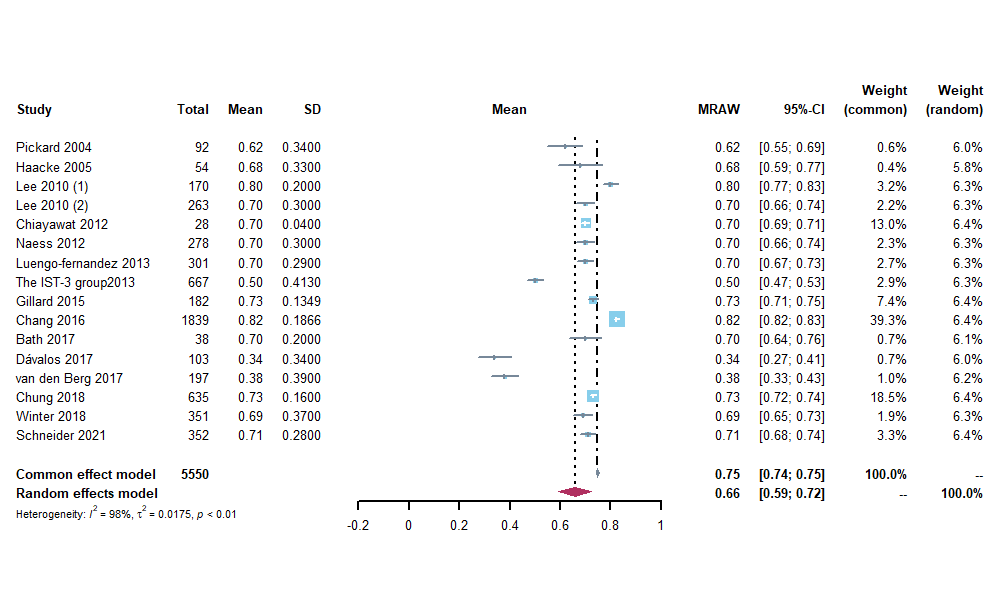
Supplementary Figure 1.** Forest plot of utilities elicited after 6 months and more poststroke.

**References**

1. Hallan S, Asberg A, Indredavik B, Widerøe TE. Quality of life after cerebrovascular stroke: a systematic study of patients' preferences for different functional outcomes. J Intern Med. 1999;246(3):309-16.

2. Pickard AS, Johnson JA, Feeny DH, Shuaib A, Carriere KC, Nasser AM. Agreement between patient and proxy assessments of health-related quality of life after stroke using the EQ-5D and Health Utilities Index. Stroke. 2004;35(2):607-12.

3. Haacke C, Althaus A, Spottke A, Siebert U, Back T, Dodel R. Long-term outcome after stroke: evaluating health-related quality of life using utility measurements. Stroke. 2006;37(1):193-8.

4. Chaiyawat P, Kulkantrakorn K, Sritipsukho P. Effectiveness of home rehabilitation for ischemic stroke. Neurol Int. 2009;1(1):e10.

5. Lee HY, Hwang JS, Jeng JS, Wang JD. Quality-adjusted life expectancy (QALE) and loss of QALE for patients with ischemic stroke and intracerebral hemorrhage: a 13-year follow-up. Stroke. 2010;41(4):739-44.

6. Chaiyawat P, Kulkantrakorn K. Effectiveness of home rehabilitation program for ischemic stroke upon disability and quality of life: a randomized controlled trial. Clin Neurol Neurosurg. 2012;114(7):866-70.

7. Naess H, Lunde L, Brogger J. The effects of fatigue, pain, and depression on quality of life in ischemic stroke patients: the Bergen Stroke Study. Vasc Health Risk Manag. 2012;8:407-13.

8. Luengo-Fernandez R, Gray AM, Bull L, Welch S, Cuthbertson F, Rothwell PM, et al. Quality of life after TIA and stroke: ten-year results of the Oxford Vascular Study. Neurology. 2013;81(18):1588-95.

9. IST-3 collaborative group. Effect of thrombolysis with alteplase within 6 h of acute ischaemic stroke on long-term outcomes (the third International Stroke Trial [IST-3]): 18-month follow-up of a randomised controlled trial. Lancet Neurol. 2013;12(8):768-76.

10. Bushnell CD, Reeves MJ, Zhao X, Pan W, Prvu-Bettger J, Zimmer L, et al. Sex differences in quality of life after ischemic stroke. Neurology. 2014;82(11):922-31.

11. Kelly ML, Rosenbaum BP, Kshettry VR, Weil RJ. Comparing clinician- and patient-reported outcome measures after hemicraniectomy for ischemic stroke. Clin Neurol Neurosurg. 2014;126:24-9.

12. Gillard PJ, Sucharew H, Kleindorfer D, Belagaje S, Varon S, Alwell K, et al. The negative impact of spasticity on the health-related quality of life of stroke survivors: a longitudinal cohort study. Health Qual Life Outcomes. 2015;13:159.

13. Alvarez-Sabín J, Santamarina E, Maisterra O, Jacas C, Molina C, Quintana M, et al. Long-Term Treatment with Citicoline Prevents Cognitive Decline and Predicts a Better Quality of Life after a First Ischemic Stroke. Int J Mol Sci. 2016;17(3):390.

14. Chang WH, Sohn MK, Lee J, Kim DY, Lee SG, Shin YI, et al. Predictors of functional level and quality of life at 6 months after a first-ever stroke: the KOSCO study. J Neurol. 2016;263(6):1166-77.

15. Rangaraju S, Frankel M, Jovin TG. Prognostic Value of the 24-Hour Neurological Examination in Anterior Circulation Ischemic Stroke: A post hoc Analysis of Two Randomized Controlled Stroke Trials. Interv Neurol. 2016;4(3-4):120-9.

16. Sand KM, Wilhelmsen G, Naess H, Midelfart A, Thomassen L, Hoff JM. Vision problems in ischaemic stroke patients: effects on life quality and disability. Eur J Neurol. 2016;23 Suppl 1:1-7.

17. Ali M, MacIsaac R, Quinn TJ, Bath PM, Veenstra DL, Xu Y,et al. Dependency and health utilities in stroke: Data to inform cost-effectiveness analyses. Eur Stroke J. 2017;2(1):70-76.

18. Bath PM, Scutt P, Blackburn DJ, Ankolekar S, Krishnan K, Ballard C, et al. Intensive versus Guideline Blood Pressure and Lipid Lowering in Patients with Previous Stroke: Main Results from the Pilot 'Prevention of Decline in Cognition after Stroke Trial' (PODCAST) Randomised Controlled Trial. PLoS One. 2017;12(1):e0164608.

19. Dávalos A, Cobo E, Molina CA, Chamorro A, de Miquel MA, Román LS, et al. Safety and efficacy of thrombectomy in acute ischaemic stroke (REVASCAT): 1-year follow-up of a randomised open-label trial. Lancet Neurol. 2017;16(5):369-376.

20. Katzan IL, Thompson NR, Lapin B, Uchino K. Added Value of Patient-Reported Outcome Measures in Stroke Clinical Practice. J Am Heart Assoc. 2017;6(7):e005356.

21. Persson J, Levin LÅ, Holmegaard L, Redfors P, Jood K, Jern C, et al. Stroke survivors' long-term QALY-weights in relation to their spouses' QALY-weights and informal support: a cross-sectional study. Health Qual Life Outcomes. 2017;15(1):150.

22. Rangaraju S, Haussen D, Nogueira RG, Nahab F, Frankel M. Comparison of 3-Month Stroke Disability and Quality of Life across Modified Rankin Scale Categories. Interv Neurol. 2017;6(1-2):36-41.

23. Schreuders J, van den Berg LA, Fransen PS, Berkhemer OA, Beumer D, Lingsma HF, et al. Quality of life after intra-arterial treatment for acute ischemic stroke in the MR CLEAN trial-Update. Int J Stroke. 2017;12(7):708-712.

24. van den Berg LA, Dijkgraaf MG, Berkhemer OA, Fransen PS, Beumer D, Lingsma HF, et al. Two-Year Outcome after Endovascular Treatment for Acute Ischemic Stroke. N Engl J Med. 2017;376(14):1341-1349.

25. Chung PW, Yoon BW, Lee YB, Shin BS, Kim HY, Park JH, et al. Medication Adherence of Statin Users after Acute Ischemic Stroke. Eur Neurol. 2018;80(1-2):106-114.

26. Dijkland SA, Voormolen DC, Venema E, Roozenbeek B, Polinder S, Haagsma JA, et al. Utility-Weighted Modified Rankin Scale as Primary Outcome in Stroke Trials: A Simulation Study. Stroke. 2018;49(4):965-971.

27. Winter Y, Daneshkhah N, Galland N, Kotulla I, Krüger A, Groppa S. Health-related quality of life in patients with poststroke epilepsy. Epilepsy Behav. 2018;80:303-306.

28. Dewilde S, Annemans L, Lloyd A, Peeters A, Hemelsoet D, Vandermeeren Y, et al. The combined impact of dependency on caregivers, disability, and coping strategy on quality of life after ischemic stroke. Health Qual Life Outcomes. 2019;17(1):31.

29. Oemrawsingh A, van Leeuwen N, Venema E, Limburg M, de Leeuw FE, Wijffels MP, et al. Value-based healthcare in ischemic stroke care: case-mix adjustment models for clinical and patient-reported outcomes. BMC Med Res Methodol. 2019;19(1):229.

30. Chen X, Wang X, Delcourt C, Li J, Arima H, Hackett ML, et al. Ethnicity and Other Determinants of Quality of Functional Outcome in Acute Ischemic Stroke: The ENCHANTED Trial. Stroke. 2020;51(2):588-593.

31. Jarosławski S, Jarosławska B, Błaszczyk B, Auqier P, Toumi M. Health-related quality of life of patients after ischaemic stroke treated in a provincial hospital in Poland. J Mark Access Health Policy. 2020;8(1):1775933.

32. Willeit P, Toell T, Boehme C, Krebs S, Mayer L, Lang C, et al. STROKE-CARD care to prevent cardiovascular events and improve quality of life after acute ischaemic stroke or TIA: A randomised clinical trial. EClinicalMedicine. 2020;25:100476.

33. Yang P, Zhang Y, Zhang L, Zhang Y, Treurniet KM, Chen W, et al. Endovascular Thrombectomy with or without Intravenous Alteplase in Acute Stroke. N Engl J Med. 2020;382(21):1981-1993.

34. Parameshwaran B, Cordato D, Parsons M, Cheung A, Manning N, Wenderoth J, et al. The Benefit of Endovascular Thrombectomy for Stroke on Functional Outcome Is Sustained at 12 Months. Cerebrovasc Dis Extra. 2021;11(2):81-86.

35. Romano JG, Gardener H, Campo-Bustillo I, Khan Y, Tai S, Riley N, et al. Predictors of Outcomes in Patients With Mild Ischemic Stroke Symptoms: MaRISS. Stroke. 2021;52(6):1995-2004.

36. Schneider S, Taba N, Saapar M, Vibo R, Kõrv J. Determinants of Long-Term Health-Related Quality of Life in Young Ischemic Stroke Patients. J Stroke Cerebrovasc Dis. 2021;30(2):105499.

37. She R, Yan Z, Hao Y, Zhang Z, Du Y, Liang Y, et al. Health-related quality of life after first-ever acute ischemic stroke: associations with cardiovascular health metrics. Qual Life Res. 2021;30(10):2907-2917.

38. Sucharew H, Kleindorfer D, Khoury JC, Alwell K, Haverbusch M, Stanton R, et al. Deriving Place of Residence, Modified Rankin Scale, and EuroQol-5D Scores from the Medical Record for Stroke Survivors. Cerebrovasc Dis. 2021;50(5):567-573.

39. Zhang P, Shen HJ, Chen L, Zhu X, Zhang MM, Jiang Y, et al. Patient-Reported Anxiety/Depression After Endovascular Thrombectomy: A post-hoc Analysis of Direct-MT Trial. Front Neurol. 2022;13:811629.
